# DFT Study of Zn-Modified SnP_3_: A H_2_S Gas Sensor with Superior Sensitivity, Selectivity, and Fast Recovery Time

**DOI:** 10.3390/nano13202781

**Published:** 2023-10-17

**Authors:** Hongyuan Cui, Chenshan Gao, Pengwei Wang, Lijie Li, Huaiyu Ye, Zhongquan Wen, Yufei Liu

**Affiliations:** 1 Key Laboratory of Optoelectronic Technology & Systems (Chongqing University), Ministry of Education, Chongqing 400044, China; hongyuan.cui@cqu.edu.cn (H.C.);; 2 Centre for Intelligent Sensing Technology, College of Optoelectronic Engineering, Chongqing University, Chongqing 400044, China; 3 School of Microelectronics, Southern University of Science and Technology, Shenzhen 518055, China; 4 Faculty of Science and Engineering, Swansea University, Swansea SA1 8EN, UK

**Keywords:** adsorption, metal-modified SnP_3_ monolayer, H_2_S sensor, DFT

## Abstract

The adsorption properties of Cu, Ag, Zn, and Cd-modified SnP_3_ monolayers for H_2_S have been studied using density functional theory (DFT). Based on phonon spectrum calculations, a structurally stable intrinsic SnP_3_ monolayer was obtained, based on which four metal-modified SnP_3_ monolayers were constructed, and the band gaps of the modified SnP_3_ monolayers were significantly reduced. The adsorption capacity of Cu, Zn-modified SnP_3_ was better than that of Ag, Cd-modified SnP_3_. The adsorption energies of Cu-modified SnP_3_ and Zn-modified SnP_3_ for H_2_S were −0.749 eV and −0.639 eV, respectively. In addition, Cu-modified SnP_3_ exhibited chemisorption for H_2_S, while Zn-modified SnP_3_ exhibited strong physisorption, indicating that it can be used as a sensor substrate. Co-adsorption studies showed that ambient gases such as N_2_, O_2_, and H_2_O had little effect on H_2_S. The band gap change rate of Zn-modified SnP_3_ after adsorption of H_2_S was as high as −28.52%. Recovery time studies based on Zn-modified SnP_3_ showed that the desorption time of H_2_S was 0.064 s at 298 K. Therefore, Zn-modified SnP_3_ can be used as a promising sensor substrate for H_2_S due to its good selectivity, sensitivity, and fast recovery time.

## 1. Introduction

With the increasing advancement of urbanization and industrialization, the domestic sewage, industrial sewage and runoff sewage gathered by pipelines produce irritant gases such as hydrogen sulfide (H_2_S) [1]. In addition, H_2_S as an industrial waste gas, its emission can pollute the atmosphere [2]. H_2_S is a highly toxic and corrosive gas, and as a kind of flammable hazardous chemical, the leakage of H_2_S may result in significant economic and property losses or even casualties. Therefore, it is critical to investigate novel solutions for detecting H_2_S gas.

With its successful discovery in 2004, graphene has demonstrated considerable promise in a variety of technical areas. Due to their substantial theoretical specific surface area, two-dimensional (2D) materials such as graphene offer excellent options for extremely sensitive sensing and detection devices [3]. However, after years of research, the sensing adsorption behavior of substrates such as transition metal dichalcogenides (TMDCs) and graphene has been systematically studied [4,5,6,7].

SnP_3_, a layered material made of Sn and P, has been studied and it was pointed out in the 1970s that it can be obtained by slow cooling to room temperature after heat treatment for 2 days at 575 °C [8]. Based on theoretical calculations, it has been found that double and single-layer SnP_3_ have relatively low cleavage energies of 0.45 J/m^2^ and 0.71 J/m^2^, separately, approached by phosphorene (0.36 J/m^2^) and graphene (0.32 J/m^2^) [9]. Therefore, SnP_3_ is a novel 2D substance that is easy to peel off. A 2D SnP_3_ not only has high thermodynamic stability [10], but also has excellent carrier mobility [11,12]. For potassium-ion, lithium-ion, and sodium-ion batteries, SnP_3_ has been used as an anode material [13,14,15]. Application studies in gas sensing have shown that intrinsic SnP_3_ has a strong adsorption effect for NO_2_ and NO [16,17,18].

In the study of gas sensing in 2D materials, computational works are frequently conducted by using density functional theory (DFT) [19,20,21]. Moreover, doped 2D materials can enhance the adsorption capacity of gases, according to DFT computational studies. Besides the impurity doping, the doping could be realized by metallic gating, and high doping carrier densities of the order of 10^14^ cm^−2^ have been achieved in 2D materials by electrolyte gating [22]. Most of the time, impurity doping could offer better selectivity, in comparison with metallic gating. For example, the interaction of O_2_ and CO is significantly enhanced by Au-doped graphene [23], As-doped WSe_2_ shows a significant increase in NO_2_ adsorption [24], and the adsorption ability of N_2_ by InN decorated with Ni atom is significantly improved [25]. However, only a few works on the doping adsorption of SnP_3_ monolayers, for example, indium-doped SnP_3_ monolayer is used for CO_2_ adsorption [26], chromium-doped SnP_3_ monolayer is used for SO_2_ adsorption [27], and palladium-doped SnP_3_ monolayer is used for H_2_ adsorption research [28].

In this work, four transition metals were selected as doping elements. Cu and Ag have similar properties because they belong to group IB. Zn and Cd have similar properties as they belong to group IIB. In addition, Cu (1.35 Å) and Zn (1.35 Å) have the same atomic radius, and Ag (1.60 Å) and Cd (1.55 Å) have similar atomic radius. By studying Cu, Ag, Zn, and Cd, the doping effects of elements in the same and adjacent groups can be compared. Therefore, the adsorption properties of H_2_S on SnP_3_ monolayers modified with Cu, Ag, Zn, and Cd atoms were studied for the first time in this paper using DFT calculations, providing theoretical support for the application of SnP_3_ monolayer material in gas sensing.

## 2. Computational Details

Herein, adsorption characteristics of the SnP_3_ monolayer were investigated using the DMol^3^ module based on DFT calculations [29,30]. The exchange correlation in the module configuration was described by the PBE (Perdew–Burke–Ernzerhof) functional in the GGA (generalized gradient approximation) [31,32]. We adjusted the dispersion interactions using the TS (Tkatchenko–Scheffler) technique (a method for DFT-D) [33,34], and checked the spin unrestricted (spin-polarization) [35]. The energy had a convergence tolerance of 10^−5^ Ha (1 Ha is approximately equal to 27.2114 eV), the maximum displacement convergence tolerance was set to 5 × 10^−3^ Å, and the convergence tolerance for maximum force was set to 2 × 10^−3^ Ha/Å [36]. DSPP (DFT Semi-core Pseudopots) was used as the core treatment technique for introducing relativistic corrections [37]. The atomic orbital used a dual numerical basis set having the orbital polarization function [38]. Smearing was set to 0.005 Ha to accelerate convergence [17]. After convergence tests, the geometric structure was optimized with a k-point set to 6 × 6 × 1, and the properties were calculated using a more intensive k-point grid of 12 × 12 × 1 [27,28]. Intrinsic and doped SnP_3_ supercell structures of 2 × 2 × 1 size with a vacuum area of 20 Å were created for adsorption studies.

After geometry optimization, the energy of the structure was obtained. The following equation was utilized to compute the adsorption energy (*E_a_*) of gas molecules on SnP_3_ substrates:

(1) $$E_{a}=E_{gas/sub}-E_{gas}-E_{sub}$$

where *E_gas/sub_* is the system’s total energy, *E_gas_* and *E_sub_* are the energies of gas molecules and substrates, respectively. The substrates are more strongly adsorbed to the gas molecules, the greater the absolute values of *E_a_*. Adsorption can typically happen on its own if the adsorption energy *E_a_* is negative.

The relevant properties were analyzed by DFT module calculations. To calculate the transfer charge (*Q*) of a gas molecule after adsorption by a substrate, Mulliken population analysis was utilized to determine the charge gain or loss of individual atoms in the gas molecule [30,39]. When *Q* is positive, the whole gas molecule consumes electrons; when *Q* is negative, the whole gas molecule accumulates electrons [40]. The shortest distance between the surface and the adsorbed gas molecule in the adsorption system was indicated by the adsorption distance (*d*). A closer adsorption distance indicates a stronger interaction, and conversely, a farther adsorption distance indicates a weaker interaction. The interactions between the substrates and the doped atoms, as well as between the substrates and the adsorbed gas molecules, were investigated through analyzing the density of states (DOS). The transfer charge between substrates and gas molecules was studied using the charge density difference (CDD). A description of whether a bond forms between the gas molecule and the substrate could be given by the electron localization function (ELF) [41,42].

## 3. Results and Discussion

### 3.1. Establishment and Analysis of SnP_3_ Monolayer

**Figure 1:**
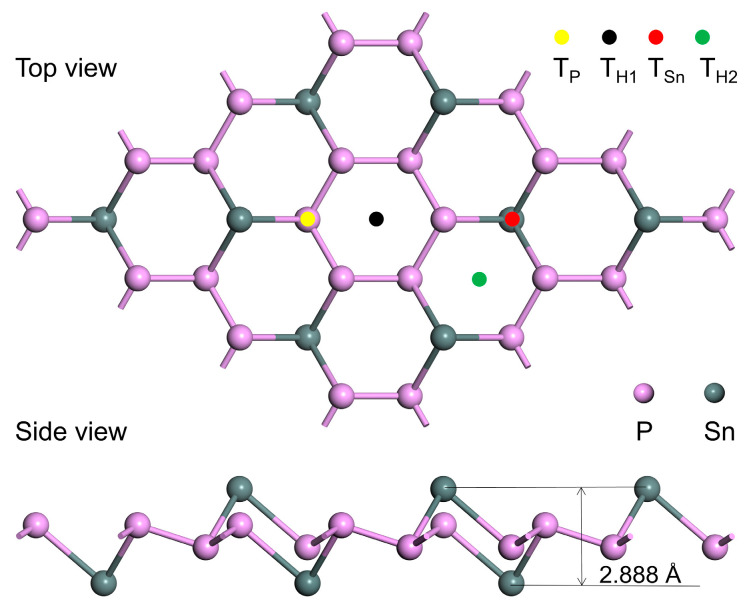
SnP_3_ monolayer structure, top and side views, with solid circles representing the modification sites.

First, based on previous research reports [27], a supercell of intrinsic SnP_3_ monolayer with a size of 2 × 2 × 1 was constructed in this paper for adsorption studies, as shown in Figure 1. The lattice parameter a = b = 7.359 Å of its unit cell is comparable to a = b = 7.37 Å in the literature [9]. The top view shows that the structure contains 8 Sn atoms and 24 P atoms. The side view shows that the structure is not flat, but a sandwich structure consisting of P atoms in the center and Sn atoms on both sides. Each P atom creates a P–Sn bond with an adjacent Sn atom and two P–P bonds with two adjacent P atoms, while three Sn–P bonds are formed between each Sn atom and the three nearby P atoms. With a thickness of 2.888 Å, the SnP_3_ monolayer is similar to that of 2.76 Å in the literature [28].

**Figure 2:**
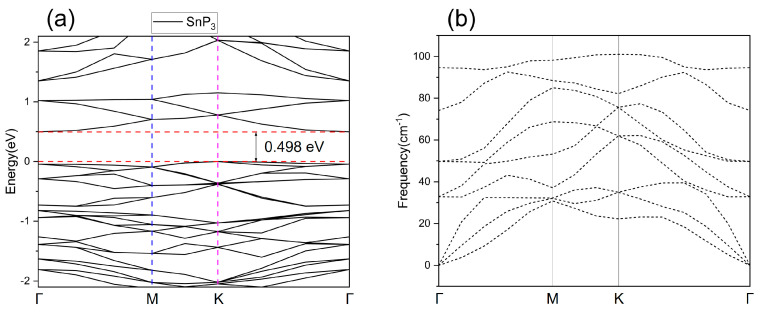
The (**a**) band structure and (**b**) phonon spectrum of the intrinsic SnP_3_ monolayer.

The intrinsic SnP_3_ band structure is shown in Figure 2a, where the horizontal coordinates are the K points taken based on the symmetry of the structural model, and the vertical coordinates indicate the energy, with the position of the zero-point corresponding to the Fermi energy level. There is a band gap of 0.498 eV for the SnP_3_ monolayer structure, which is consistent with the findings of earlier research [31,43]. To verify the stability of the constructed monolayer structure, we calculated the phonons using the finite displacement method in the CASTEP module. The same functional and DFT-D corrections as in the DMol^3^ were used in the module setup, and additionally, an energy cutoff of 500 eV and ultrasoft pseudopotentials were set. The phonon spectrum can reflect whether the material exists stably or not, if the phonon spectrum of the material has no imaginary frequency, it can be proved that the material can exist stably. As shown in Figure 2b, there is no imaginary frequency in the phonon spectrum of the intrinsic SnP_3_ structure, which can be a good indication that the structure is stable. On this basis, we will construct several metal doped structures.

Next, the modified doping model was constructed based on intrinsic SnP_3_. Four transition metal atoms, Cu, Ag, Zn, and Cd were selected as doping elements. As depicted in Figure 1, four distinct modification locations were taken into account in the construction of the metal atom doping structure, namely, the T_P_ location above the P atom, the T_H1_ location above the hexagonal ring composed of P atoms, the T_Sn_ location above the Sn atom, and the T_H2_ location above the hexagonal ring made up of P and Sn atoms. Usually, the stability of the doped structure can be evaluated by calculating the formation energy [44], and the formation energy (*E_f_*) can be calculated according to the following equation:

(2) $$E_{f}=E_{X-SnP3}-E_{SnP3}-E_{X}$$

where *E_X_*_–*SnP*3_, *E_SnP_*_3_ and *E_X_* are the energies of the X-modified SnP_3_ (X = Cu, Ag, Zn, Cd), SnP_3_, and a metal atom, respectively. The more negative the formation energy, the more stable the doped structure. It can be seen that the energy of the doped structure determines the formation energy, since the energy of SnP_3_ and metal atoms are certain. After comparison, the doped structure with the metal atom modified above the Sn atom has the maximum absolute value of energy, that is, the most stable structure, so the T_Sn_ site is the best modification site, which is consistent with the doping site in the literature [28].

**Figure 3:**
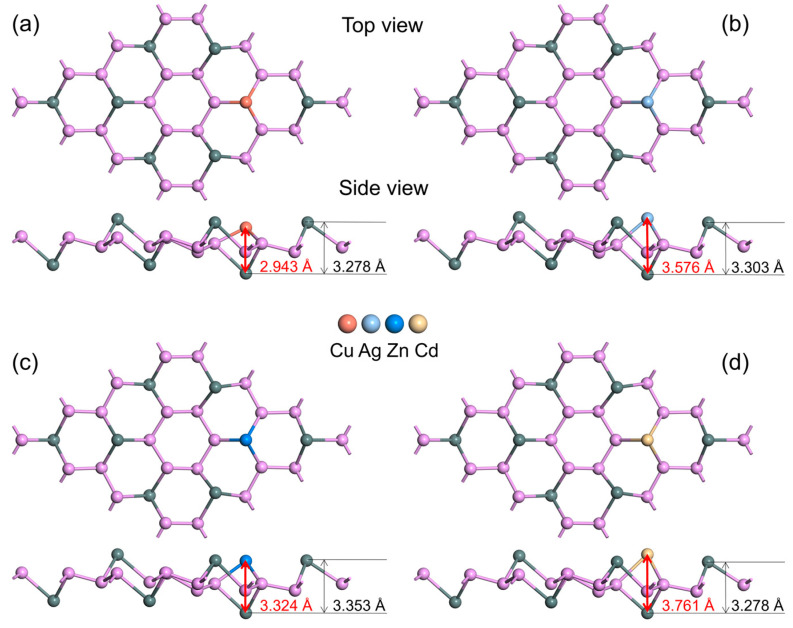
Optimized structures of (**a**) Cu–SnP_3_, (**b**) Ag–SnP_3_, (**c**) Zn–SnP_3_, and (**d**) Cd–SnP_3_.

The stable structures of the SnP_3_ monolayers modified by four metal atoms (Cu–SnP_3_, Ag–SnP_3_, Zn–SnP_3_, Cd–SnP_3_) are shown in Figure 3. The average lengths of the Cu–P, Ag–P, Zn–P, and Cd–P bonds formed by the modified metal atoms with three P atoms are 2.275 Å, 2.622 Å, 2.480 Å, and 2.812 Å, respectively. In addition, the distances of the modified metal atoms from the Sn atoms directly below them are 2.943 Å, 3.576 Å, 3.324 Å, and 3.761 Å, respectively. Compared with the intrinsic SnP_3_ monolayer, the thickness of doped SnP_3_ monolayers increased to 3.278 Å, 3.303 Å, 3.353 Å, and 3.278 Å, respectively.

**Figure 4:**
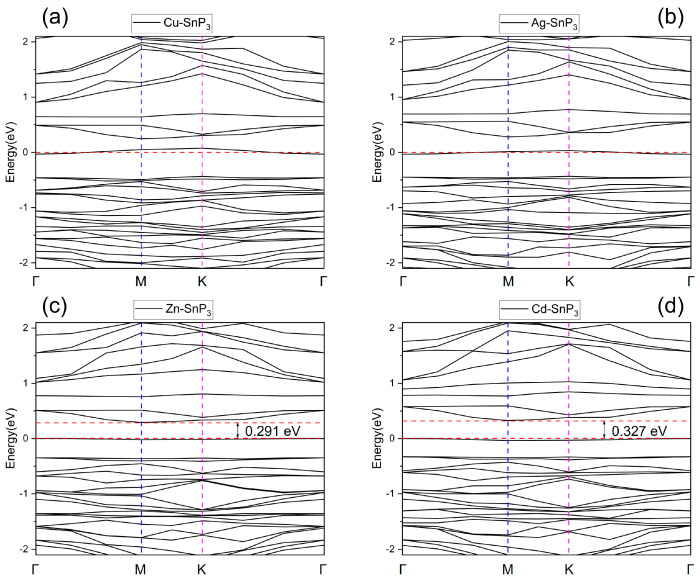
Energy band structures of (**a**) Cu–SnP_3_, (**b**) Ag–SnP_3_, (**c**) Zn–SnP_3_, and (**d**) Cd–SnP_3_.

The modified SnP_3_ monolayer energy band structures are shown in Figure 4. The band gap of intrinsic SnP_3_ is 0.498 eV. The band gap of the atom-doped SnP_3_ monolayer is reduced, and the energy bands of Cu–SnP_3_ and Ag–SnP_3_ cross the Fermi energy level and exhibit metallic properties, and the band gaps of Zn–SnP_3_ and Cd–SnP_3_ are 0.291 eV and 0.327 eV, respectively.

**Figure 5:**
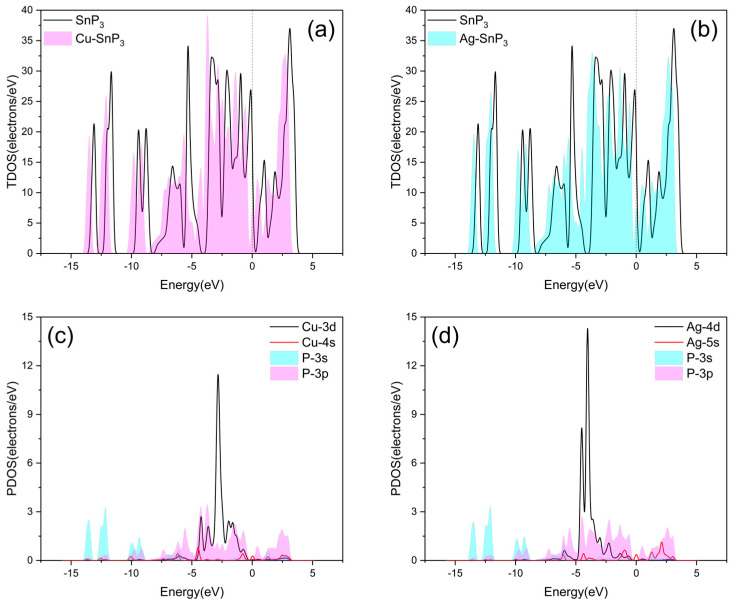
TDOS of (**a**) Cu–SnP_3_ and (**b**) Ag–SnP_3_. PDOS of (**c**) Cu–SnP_3_ and (**d**) Ag–SnP_3_.

**Figure 6:**
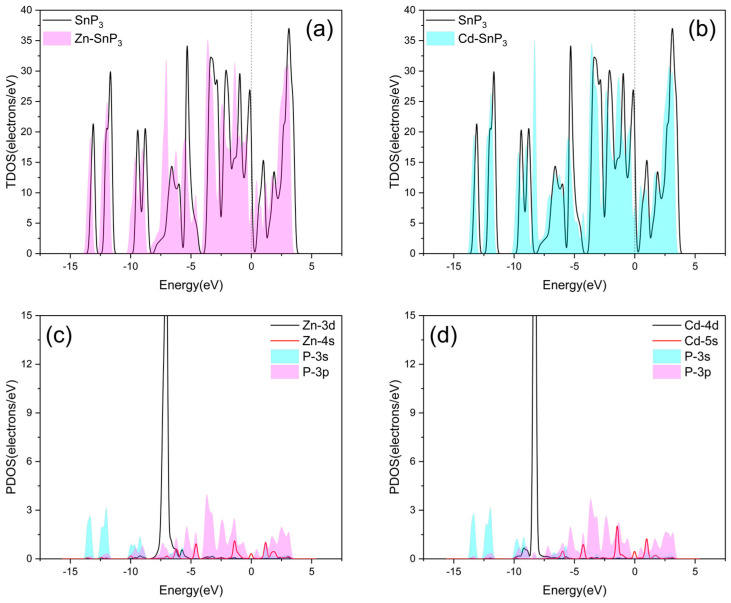
TDOS of (**a**) Zn–SnP_3_ and (**b**) Cd–SnP_3_. PDOS of (**c**) Zn–SnP_3_ and (**d**) Cd–SnP_3_.

The DOS of intrinsic and four doped SnP_3_ monolayers are depicted in Figure 5 and Figure 6. The zero point of the energy coordinate indicates the Fermi energy level. Below zero is the valence band, and all positions are filled with electrons. The material properties have a strong correlation with the energy band structure close to the Fermi energy level. By studying the TDOS (total DOS) and the PDOS (projected DOS), we analyzed the effect of Cu, Ag, Zn, and Cd doping on the SnP_3_ monolayer, and the interactions between the SnP_3_ monolayer and the doped transition metal atoms.

Figure 5a,b and Figure 6a,b illustrate the TDOS of four doped SnP_3_ monolayers compared with the intrinsic SnP_3_, respectively. It is obvious that the TDOS of the doped SnP_3_ monolayers is shifted to the left with respect to the intrinsic monolayer, and combined with the energy band structure in Figure 4, indicates that doping reduces the band gap, which results in enhanced metal characteristics and increased conductivity. Moreover, as the band gap narrows, the valence band electrons can move more readily into the conduction band [45].

The PDOS of Cu–SnP_3_ is seen in Figure 5c. The P-3p and Cu-3d orbits significantly superimpose between −4.682 eV and −0.361 eV. It shows that the P-3p and Cu-3d orbitals have strong interactions and orbital hybridization mostly happens between them, which will cause the electron distribution to alter, and denotes the formation of the Cu–P bond. It demonstrates that the Cu atom modified on the SnP_3_ monolayer surface has a stable structure. The PDOS of Ag–SnP_3_ is shown in Figure 5d. It is clear that the Ag-4d and P-3p orbits significantly overlap between −4.831 eV and −1.913 eV, and the Ag-5s and P-3p orbits overlap slightly at the peak of 2.082 eV. It shows that Ag-4d, Ag-5s, and P-3p orbitals have strong interactions. Orbital hybridization mostly happens between Ag-4d and P-3p, which will cause the electron distribution to alter, and indicates the formation of the Ag–P bond. It shows that the Ag atom modified on the SnP_3_ monolayer surface has a stable structure.

The PDOS of Zn–SnP_3_ is depicted in Figure 6c. The Zn-3d and P-3s, P-3p orbits overlap between −8.374 eV and −4.913 eV, and the Zn-4s and P-3p orbital peaks overlap at −4.580 eV, −1.212 eV, and 1.151 eV. It indicates that all orbitals interact with each other. Orbital hybridization mostly happens between Zn-4s and P-3p, which will cause the electron distribution to alter, and indicates the formation of the Zn–P bond. It shows that the Zn atom modified on the SnP_3_ monolayer surface has a stable structure. The PDOS of Cd–SnP_3_ is seen in Figure 6d. The Cd-4d and P-3s, P-3p orbits overlap between −9.852 eV and −7.281 eV, and the peaks of the Cd-5s and P-3p orbits coincide at −4.276 eV, −1.456 eV, and 0.973 eV, which means there are interactions between the orbitals. Orbital hybridization mostly happens between P-3p and Cd-5s, which will cause the electron distribution to alter, and indicates the formation of the Cd–P bond. It shows that the Cd atom modified on the SnP_3_ monolayer surface has a stable structure.

### 3.2. Study on Adsorption of H_2_S by SnP_3_ Monolayer

In constructing the intrinsic SnP_3_ monolayer adsorption system for H_2_S, the initial separation between the H_2_S and the SnP_3_ monolayer was adjusted to 3 Å. As shown in Figure S1, various adsorption positions of the H_2_S molecule are considered, including H_2_S molecules placed parallel or vertically above the Sn atom, P atom, and the hexagonal ring. According to modular calculations, the intrinsic SnP_3_ monolayer adsorbed H_2_S has the most stable structure, as illustrated in Figure S1b. Similarly, the system of X–SnP_3_ (X = Cu, Ag, Zn, Cd) for H_2_S was constructed using the above method. Since the doped metal atoms act as interacting active sites between the substrate and the adsorbed gases [46], the H_2_S molecule was placed parallel or vertically on the doped metal atoms in the construction of the adsorption systems of the four doped SnP_3_ monolayers to H_2_S.

**Figure 7:**
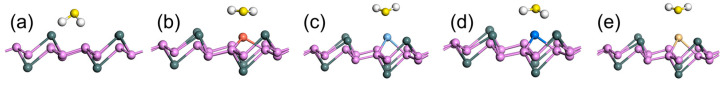
Optimized structures of (**a**) intrinsic SnP_3_, (**b**) Cu–SnP_3_, (**c**) Ag–SnP_3_, (**d**) Zn–SnP_3_, and (**e**) Cd–SnP_3_ for H_2_S adsorption.

As shown in Figure 7, the most stable structures of the intrinsic as well as the four doped SnP_3_ monolayers for H_2_S adsorption are displayed. Table 1 summarizes the results of H_2_S adsorbed on the intrinsic SnP_3_ and the SnP_3_ doped with four metal atoms. It includes the adsorption energy (*E_a_*) of the SnP_3_ monolayer to the H_2_S molecule, the transfer charge (*Q*) of the H_2_S molecule, and the shortest adsorption distance (*d*) between the SnP_3_ monolayer and the H_2_S. According to Equation (1), the adsorption energy of intrinsic SnP_3_ for H_2_S is −0.392 eV. The transfer charge and adsorption distance are 0.024 e and 2.574 Å, respectively. The X–SnP_3_ (X = Cu, Ag, Zn, Cd) monolayer adsorption energies for H_2_S are −0.749 eV, −0.595 eV, −0.639 eV, and −0.402 eV, correspondingly. The absolute values of adsorption energies are Cu–SnP_3_ > Zn–SnP_3_ > Ag–SnP_3_ > Cd–SnP_3_. The transfer charges of H_2_S molecules amounted to 0.272 e, 0.211 e, 0.234 e, and 0.187 e, correspondingly. The transfer charges show numerically Cu–SnP_3_ > Zn–SnP_3_ > Ag–SnP_3_ > Cd–SnP_3_. The adsorption distances, which are 2.336 Å, 2.601 Å, 2.520 Å, and 2.877 Å, showed numerically that Cu–SnP_3_ < Zn–SnP_3_ < Ag–SnP_3_ < Cd–SnP_3_.

Compared with the intrinsic SnP_3_ monolayer, the transfer charge and adsorption energy values of the four doped SnP_3_ monolayers are increased, and the adsorption distances between Cu–SnP_3,_ Zn–SnP_3_, and H_2_S molecule are smaller than those of the intrinsic SnP_3_ monolayer. The larger the value of the transfer charge and adsorption energy, and the closer the adsorption distance, the better the adsorption effect is considered. Therefore, based on the information in Table 1, the SnP_3_ monolayer doped with four kinds of metals can improve the adsorption of H_2_S, only Cu–SnP_3_ and Zn–SnP_3_ can significantly improve the adsorption of H_2_S, while Ag–SnP_3_ and Cd–SnP_3_ have a smaller enhancing effect on the adsorption of H_2_S.

Next, to study the effects of adsorbed H_2_S on the electronic structure of the SnP_3_ monolayers, DOS, CDD, and ELF analyses were performed for the adsorption system. Since Cu–SnP_3_ and Zn–SnP_3_ have better adsorption capacities for H_2_S than Ag–SnP_3_ and Cd–SnP_3_, here we only took Cu–SnP_3_ and Zn–SnP_3_ as examples to study the adsorption of H_2_S.

**Figure 8:**
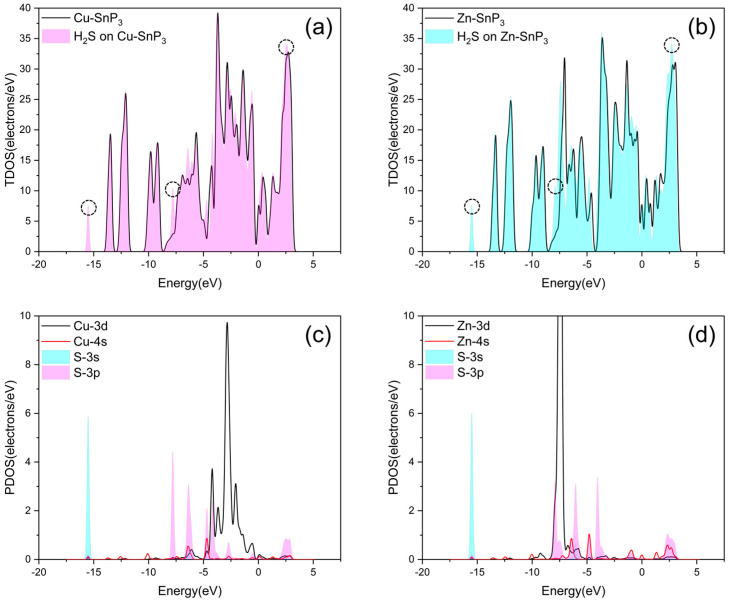
TDOS after adsorption of H_2_S by (**a**) Cu–SnP_3_ and (**b**) Zn–SnP_3_. PDOS after adsorption of H_2_S by (**c**) Cu–SnP_3_ and (**d**) Zn–SnP_3_.

As illustrated in Figure 8a,b, the TDOS of Cu–SnP_3_ and Zn–SnP_3_ monolayers is not significantly shifted after H_2_S adsorption, but there is an increase at some positions (marked by circles), and the increased positions correspond exactly to the S-3s, 3p orbitals of the PDOS in Figure 8c,d. From Figure 8c, the Cu-3d, 4s, and S-3p orbital peaks overlap at −6.362 eV, −4.708 eV, −4.197 eV, −2.762 eV, and 2.663 eV. The overlap of the same waveform of the density of states peak represents the hybridization between the adsorbed molecule and the substrate, thus indicating a strong interaction of the S atom in H_2_S with the substrate-modified Cu atom. In addition, as seen in Figure 8d, the Zn-3d, 4s, and S-3p orbital peaks overlap at −7.465 eV, −6.069 eV, −4.745 eV, −4.065 eV, and 2.528 eV, suggesting that the doped Zn atom interacts strongly with the S atom in the H_2_S molecule.

**Figure 9:**
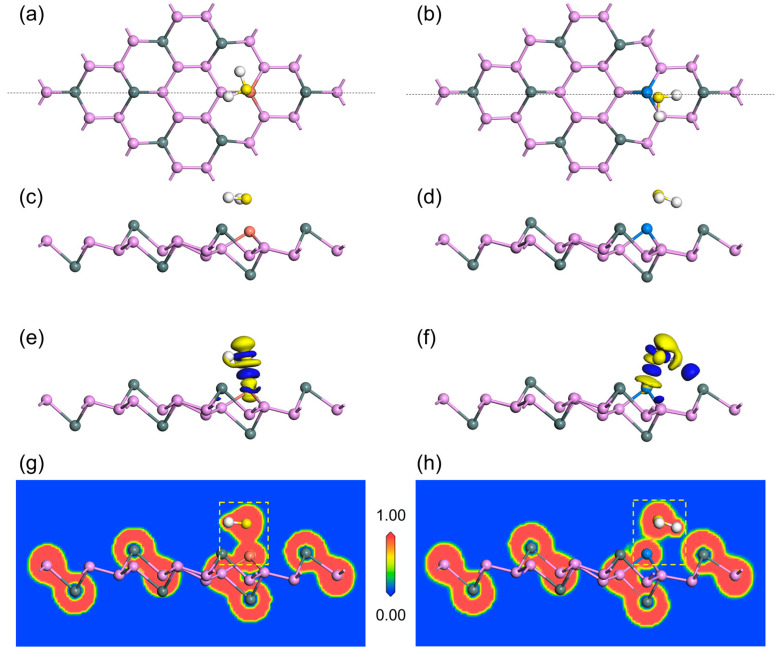
Top view of (**a**) H_2_S/Cu–SnP_3_ and (**b**) H_2_S/Zn–SnP_3_. Side view of (**c**) H_2_S/Cu–SnP_3_ and (**d**) H_2_S/Zn–SnP_3_. CDD of (**e**) H_2_S/Cu–SnP_3_ and (**f**) H_2_S/Zn–SnP_3_. ELF of (**g**) H_2_S/Cu–SnP_3_ and (**h**) H_2_S/Zn–SnP_3_.

Figure 9a–d shows the top and side perspectives of Cu–SnP_3_ and Zn–SnP_3_ structures that are most stable to H_2_S adsorption, respectively. The CDD of H_2_S adsorbed by Cu–SnP_3_ and Zn–SnP_3_ is seen in Figure 9e,f. In the study of adsorption, CDD can be used to observe the direction of charge transfer in space after molecular adsorption. The isosurface can be obtained by connecting the points with the same charge gain and loss probability. Here, we set the isosurface value to 0.015 e/Å^3^, which can obtain a better graphical effect. The yellow and blue parts of the CDD illustration indicate charge consumption and accumulation, respectively. The charge of H atoms inside the H_2_S molecule is consumed, while the charge of the S atom is accumulated. However, the charge density of the H_2_S molecule is reduced from the overall view of the adsorption system, indicating that the substrate receives the charge from H_2_S. As a comparison, the CDD of the intrinsic SnP_3_ adsorbed H_2_S is displayed in Figure S2c, showing little transfer charge between the substrate and H_2_S molecule. From Table 1, the charge transfer between H_2_S and Cu–SnP_3_, and Zn–SnP_3_ was calculated by Mulliken population analysis to be 0.272 e, and 0.234 e, separately. The positive transfer charge value shows that the H_2_S molecule is positively charged, which implies that the H_2_S molecule loses electrons and transfers to the substrate, so the population analysis value is consistent with the CDD result.

Electron localization function (ELF) is one of the means to study electronic structure, which can characterize the localization degree of electrons. ELF is used to show the distribution of electrons outside the nucleus and analyze the properties of electrons near the nucleus and bonding regions. The range of the ELF value is 0 to 1: when ELF = 1, the electron is completely localized; when ELF = 0, it corresponds to complete delocalization of the electron, which also means that there is no electron at that place [47]. Figure 9g,h formed along the lines in Figure 9a,b show the ELF section plots of Cu–SnP_3_ and Zn–SnP_3_ adsorbing H_2_S. In the ELF diagram, the closer the blue area is, the less and more dispersed the electrons are. The closer the red area is, the more concentrated the electrons are. From the dashed box in Figure 9g, the overlap of electron localization between the doped Cu atom and the S atom in H_2_S, indicates the existence of shared electrons and the formation of a chemical bond between them, suggesting that Cu–SnP_3_ is chemically adsorbed to H_2_S. As shown in Figure 9h, it is clear from the dotted box that the Zn atom and the S atom electron localization edges are very close to each other with strong interactions, indicating that Zn–SnP_3_ is a strong physisorption for H_2_S. As a comparison, the ELF of H_2_S adsorption by intrinsic SnP_3_ is shown in Figure S2d, which shows that the electron localization is not overlapped between the H_2_S molecule and the SnP_3_ and is far away, indicating that intrinsic SnP_3_ has only weak physical adsorption of H_2_S.

### 3.3. Study on Co-Adsorption of H_2_S with Ambient Gases

**Figure 10:**
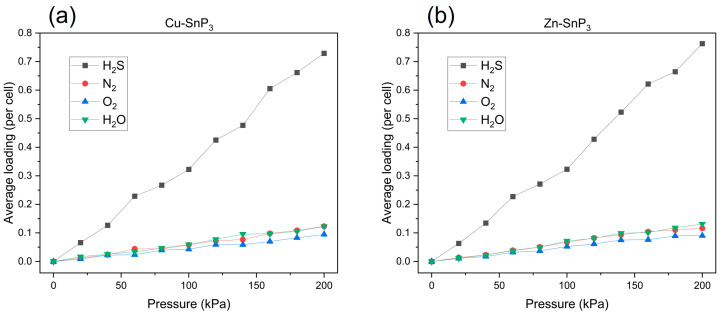
Adsorption isotherms of H_2_S, N_2_, O_2_, and H_2_O on (**a**) Cu–SnP_3_ and (**b**) Zn–SnP_3_ at pressures ranging from 0 to 200 kPa and at temperatures of 298 K.

To study the interference of ambient gases with H_2_S, we calculated isotherm adsorption curves of the SnP_3_ monolayer for H_2_S as well as for the three interfering gases (N_2_, O_2_, and H_2_O) using the Metropolis method in the Sorption module. Cu–SnP_3_ and Zn–SnP_3_ were chosen as the adsorption substrates because of their better adsorption capacity for H_2_S than Ag–SnP_3_ and Cd–SnP_3_. As shown in Figure 10, the horizontal axis represents the pressure, and the vertical axis represents the adsorption capacity. When the pressure is between 0 and 200 kPa and the temperature is 298 K, the adsorption capacity of the substrate for several gases improves with increasing pressure. Notably, the adsorption of H_2_S by the substrate was significantly greater than that of N_2_, O_2_, and H_2_O throughout the co-adsorption process. At pressures ranging from 20 to 200 kPa, the adsorption capacity of Cu–SnP_3_ and Zn–SnP_3_ for H_2_S was approximately six to seven times that of N_2_, O_2_, and H_2_O. The results showed that Cu–SnP_3_ and Zn–SnP_3_ had good selectivity for H_2_S, which provided a strong theoretical support for the adsorption of H_2_S in the ambient environment.

### 3.4. Study on Sensing of H_2_S by Zn–SnP_3_

**Figure 11:**
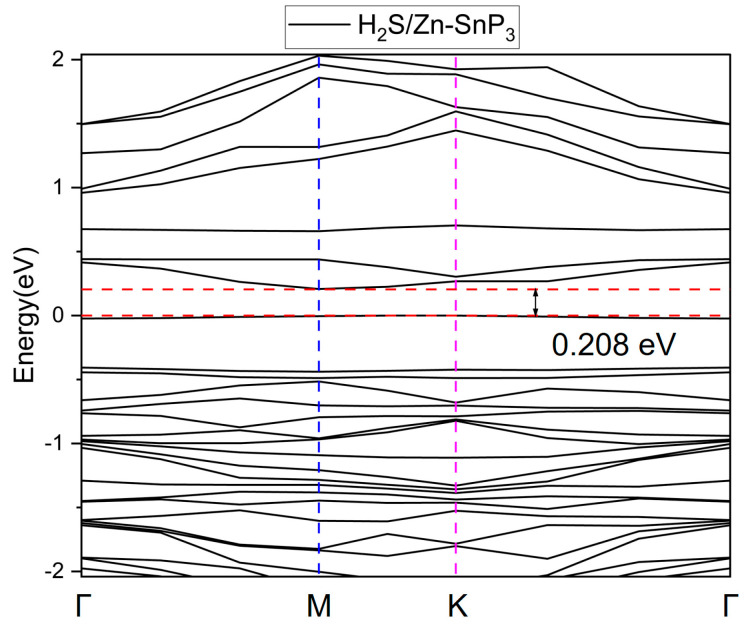
Band structure of Zn–SnP_3_ after adsorption of H_2_S.

The above studies have shown that Zn–SnP_3_ has strong physical adsorption on H_2_S, therefore Zn–SnP_3_ is a potentially sensitive material for the detection of H_2_S. Sensor devices can be fabricated from Zn–SnP_3_ materials and applied to the detection of the target gas H_2_S. Typically, when a sensitive material adsorbs a target substance, the electrical conductivity of that material changes at the macro level. The conductivity (*σ*) can be described by the following relation [48]:

(3) $$\sigma \propto \exp\left(\frac{-E_{g}}{2TK_{B}}\right)$$

where *E_g_* in the relation represents the band gap, and *T* and *K_B_* are the temperature and Boltzmann constant, respectively. It is easy to notice from this relation that only the parameter *E_g_* affects the conductivity of the adsorption system at a certain temperature. The change in band gap after gas adsorption directly affects the change in conductivity, and the more obvious the change in conductivity, the easier the signal of current or voltage in the device can be detected. The band gap of Zn–SnP_3_ is 0.291 eV, as shown in Figure 4. The energy band structure of Zn–SnP_3_ after adsorption of H_2_S is shown in Figure 11, with the band gap of 0.208 eV.

The adsorption energy, transfer charge, band gap, and band gap change rate of H_2_S adsorbed by different substrate materials were summarized, as shown in Table 2. It can be seen from the table that compared with the intrinsic substrate, the absolute values of adsorption energy and transfer charge of H_2_S on doped substrate have increased, indicating the enhancement of adsorption performance. However, it does not mean that these doped substrate materials can be used as sensitive materials for sensors. For example, after adsorption of H_2_S by Zn–MoSe_2_, the band gap change rate was −7.06%, indicating its low sensitivity to H_2_S, but due to its larger adsorption energy (−1.361 eV), it can be applied to gas elimination [49]. In this work, the band gap change rate of Zn–SnP_3_ after adsorption of H_2_S is as high as −28.52%, significantly higher than that of other references, which strongly suggests that Zn–SnP_3_ can be used as a sensitive material for H_2_S.

**Figure 12:**
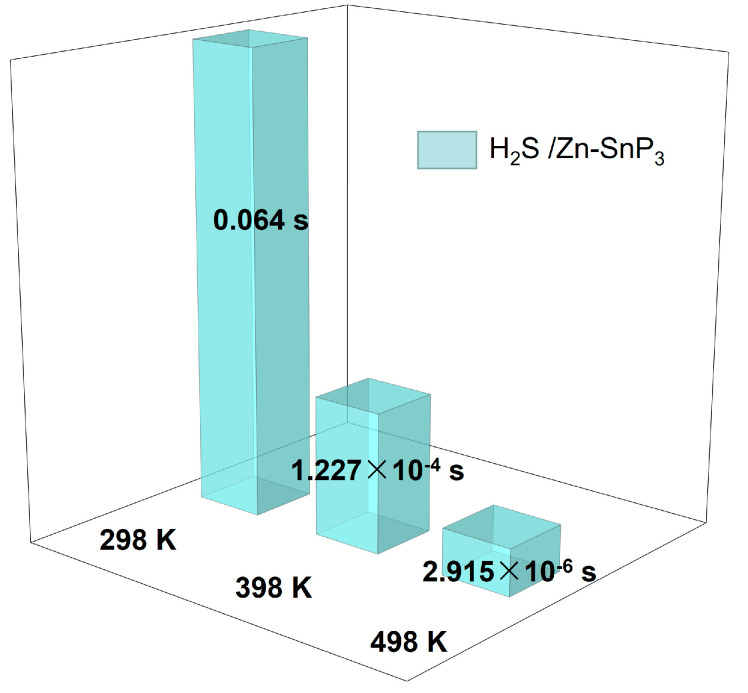
The recovery time of H_2_S at 298 K, 398 K, and 498 K, respectively.

In addition, the recovery time is one of the important indicators of the gas sensor. Generally, a good gas sensor should have a short recovery time, and the recovery time (*τ*) can be estimated from Van’t Hoff–Arrhenius expression for the rate constant, which can be calculated by the following definition [51]:

(4) $$\tau =\gamma^{-1}\exp\left(-E_{a}/TK_{B}\right)$$

where according to transition state theory the attempt frequency *γ* is commonly assumed to be ‘a typical value’ in the range of 10^12^–10^13^ s^−1^ [52], which is taken here to be 10^12^ s^−1^. *E_a_* represents the adsorption energy with the values shown in Table 1. *T* is the temperature, in K. *K_B_* denotes the Boltzmann constant with a value of 8.62 × 10^−5^ eV K^−1^. The recovery time increases with the absolute value of the adsorption energy and decreases with the temperature. The recovery time of the H_2_S gas molecule at different temperatures is shown in Figure 12. The desorption time at 298 K is only 0.064 s, indicating that Zn–SnP_3_ has a fast recovery rate for H_2_S at room temperature. As a comparison, the recovery time after H_2_S adsorption on Cu–SnP_3_ was calculated, as shown in Figure S3, which shows that the desorption time of H_2_S adsorbed on Cu–SnP_3_ is significantly longer than that of Zn–SnP_3_. In addition, the attempt frequency *γ* can be associated with the vibrational frequency [51]. Some studies have shown that light exposure is related to gas desorption [53,54], which may be due to the fact that different wavelengths of light change the vibrational frequency, which in turn has an effect on the attempt frequency and alters the recovery time.

## 4. Conclusions

Overall, this work has used the density functional theory (DFT) to study in detail the adsorption of intrinsic SnP_3_ and metal atom modified SnP_3_ to irritant H_2_S gases. The monolayers of X–SnP_3_ (X = Cu, Ag, Zn, Cd) with stable structures were constructed, and all four atoms were modified at the same position, showing good consistency. The intrinsic SnP_3_ monolayer showed only physical adsorption and weak interaction for H_2_S, while the four doped monolayers showed enhanced adsorption capacity for H_2_S. Because the adsorption capacity of Cu–SnP_3_ and Zn–SnP_3_ is better than that of Ag–SnP_3_ and Cd–SnP_3_, the adsorption mechanism of Cu–SnP_3_ and Zn–SnP_3_ monolayer to H_2_S was further investigated. The findings indicate that the strong interactions between gas molecules and Cu–SnP_3_ and Zn–SnP_3_ monolayers result from the hybridization of orbitals and transfer of charges between gas molecules and modified Cu and Zn atoms. The Cu–SnP_3_ monolayer shows chemical adsorption for H_2_S, while the Zn–SnP_3_ monolayer shows physical adsorption with a strong interaction for H_2_S. Co-adsorption studies of ambient gases (N_2_, O_2_, and H_2_O) with H_2_S showed that SnP_3_ has good selectivity for H_2_S. The study of recovery time showed that at 298 K, 398 K, and 498 K, the desorption time of H_2_S was 0.064 s, 1.227 × 10^−4^ s, and 2.915 × 10^−6^ s. It is indicated that H_2_S has a rapid desorption ability at room temperature. In this work, the Zn–SnP_3_ monolayer was developed based on metal doping with good selectivity and sensitivity to H_2_S, which can provide theoretical support for the application of 2D materials in gas sensing.

## Figures and Tables

**Table 1:** Parameters of H_2_S molecule adsorption by five SnP_3_ substrates. *E_a_*, *Q*, and *d* stand for the adsorption energy, transfer charge, and shortest adsorption distance, respectively.

System	*E_a_* (eV)	*Q* (e)	*d* (Å)
H_2_S/SnP_3_	−0.392	0.024	2.574 (P–H)
H_2_S/Cu–SnP_3_	−0.749	0.272	2.336 (Cu–S)
H_2_S/Ag–SnP_3_	−0.595	0.211	2.601 (Ag–S)
H_2_S/Zn–SnP_3_	−0.639	0.234	2.520 (Zn–S)
H_2_S/Cd–SnP_3_	−0.402	0.187	2.877 (Cd–S)

**Table 2:** Summary of adsorption energy (*E_a_*), transfer charge (*Q*), band gap of the substrate before and after adsorption of the gases (*E_g_*_1_ and *E_g_*_2_), change in band gap (Δ*E_g_* = *E_g_*_2_ − *E_g_*_1_), and rate of change in band gap (Δ*E_g_*/*E_g_*_1_) for H_2_S adsorption on different substrates.

Substrate	*E_a_* (eV)	*Q* (e)	*E_g_*_1_ (eV)	*E_g_*_2_ (eV)	Δ*E_g_* (eV)	Δ*E_g_*/*E_g_*_1_	Reference
MoSe_2_	−0.250	−0.063	1.609	1.605	−0.004	−0.25%	[49]
SnP_3_	−0.363	0.050	0.546	0.543	−0.003	−0.5%	[17]
MoS_2_	/	0	2.06	2.03	−0.03	−1.5%	[50]
Zn–MoSe_2_	−1.361	−0.323	0.326	0.303	−0.023	−7.06%	[49]
Zn–SnP_3_	−0.639	0.234	0.291	0.208	−0.083	−28.52%	This work

## Data Availability

The data will be furnished upon reasonable request.

## Supplementary Materials

The following supporting information can be downloaded at: https://www.mdpi.com/article/10.3390/nano13202781/s1.
