# Supplementary material for: DFT Study of Zn-Modified SnP3: A H2S Gas Sensor with Superior Sensitivity, Selectivity, and Fast Recovery Time
Source: Nanomaterials (Basel). 2023 Oct 17;13(20):2781. doi: 10.3390/nano13202781 (PMC10609550; doi:10.3390/nano13202781)
Supplement: Supplementary file 1 [file nanomaterials-13-02781-s001.zip › nanomaterials-2655356-supplementary.pdf]

Supporting Information

DFT Study of Zn-Modified SnP_3: A H_2S Gas Sensor with Superior Sensitivity, Selectivity, and Fast Recovery Time

Hongyuan Cui[a,b], Chenshan Gao[c], Pengwei Wang[a,b], Lijie Li[d], Huaiyu Ye[c], Zhongquan Wen[a], Yufei Liu[a,b,d,*]

[a] Key Laboratory of Optoelectronic Technology & Systems (Chongqing University), Ministry of Education, Chongqing 400044, China
[b] Centre for Intelligent Sensing Technology, College of Optoelectronic Engineering, Chongqing University, Chongqing 400044, China
[c] School of Microelectronics, Southern University of Science and Technology, Shenzhen 518055, China
[d] Faculty of Science and Engineering, Swansea University, Swansea SA1 8EN, UK

[*]To whom correspondence should be addressed
Key Laboratory of Optoelectronic Technology & Systems (Chongqing University),
Ministry of Education, Chongqing 400044, China
E-mail address: Yufei.Liu@cqu.edu.cn

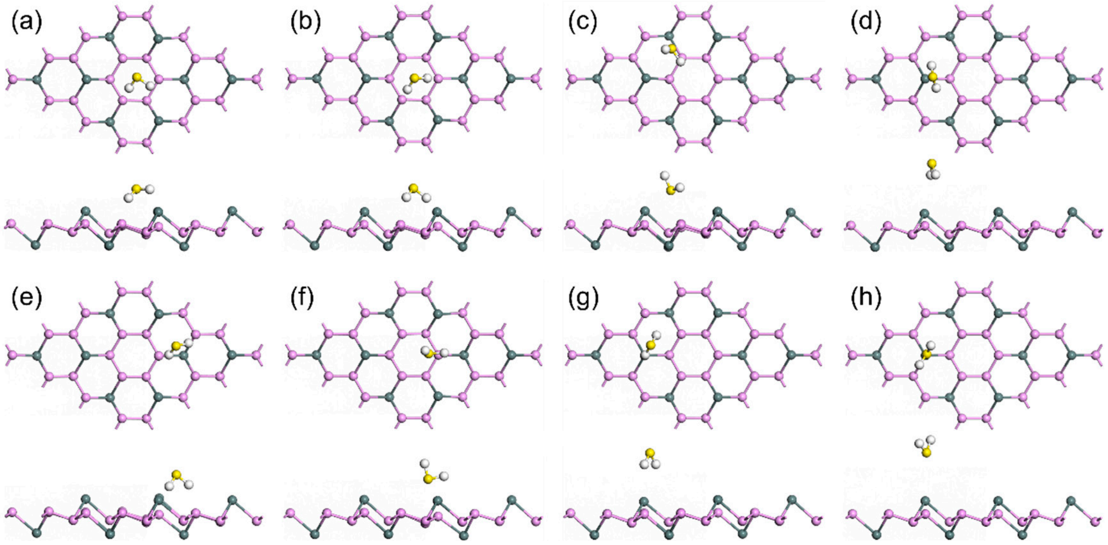

Fig. S1. Multiple optimized structures of intrinsic SnP_3 monolayer for H_2S adsorption.
(a) H_2S is horizontally adsorbed on the P atom (P-H).
(b) H_2S is horizontally adsorbed on the P atom (P-S).
(c) H_2S is horizontally adsorbed on the Sn atom (Sn-H).
(d) H_2S is horizontally adsorbed on the Sn atom (Sn-S).
(e) H_2S is vertically adsorbed on the P atom (P-H).
(f) H_2S is vertically adsorbed on the P atom (P-S).
(g) H_2S is vertically adsorbed on the Sn atom (Sn-H).
(h) H_2S is vertically adsorbed on the Sn atom (Sn-S).
Atom color code: H–white, S–yellow, Sn–grey, and P–purple.

Fig. S1 shows a variety of optimized structures of intrinsic SnP_3 after H_2S adsorption. Horizontal adsorption is the placement of the H_2S molecule horizontally above the SnP_3 monolayer and is divided into two cases: H-atom alignment and S-atom alignment. Vertical adsorption is the placement of the H_2S molecule vertically above the SnP_3 monolayer and is divided into two cases: H-atom alignment and S-atom alignment.

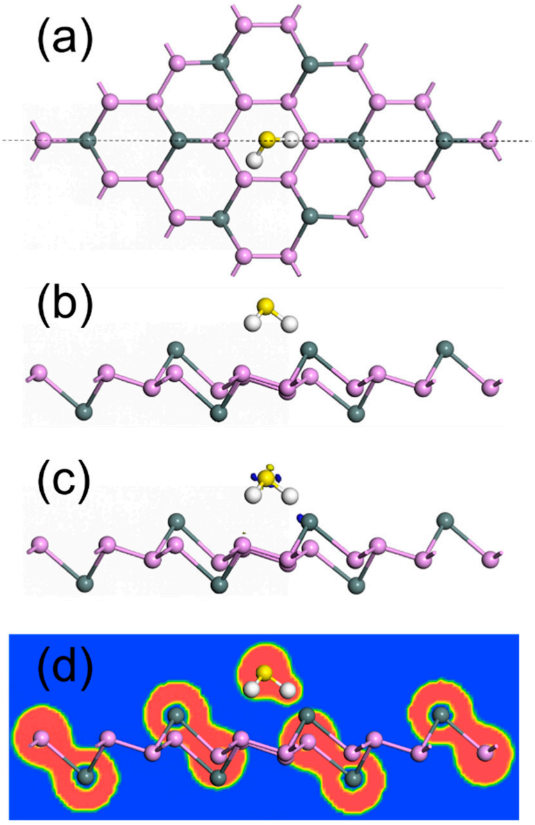

Fig. S2. (a)-(b) Top and side views of the most stable structure of intrinsic SnP_3 monolayer adsorbed H_2S, (c) charge density difference (CDD), (d) electron localization function (ELF).

Fig. S2(a) and S2(b) show the top and side views of the most stable structure of the intrinsic SnP_3 monolayer adsorbed H_2S (as in the case of Fig. S1(b)). Fig. S2(c) and S2(d) show the CDD and ELF, respectively.

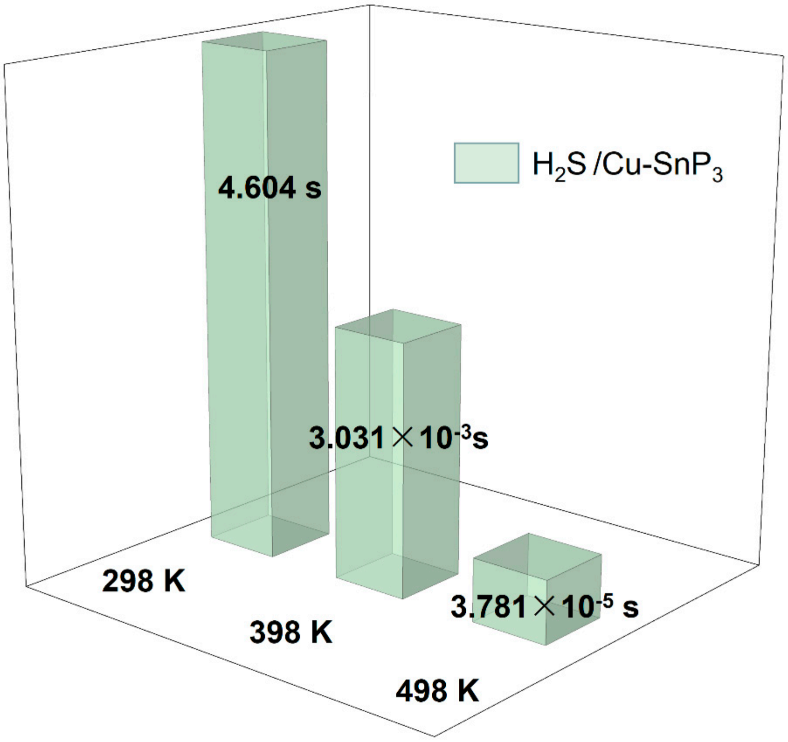

Fig. S3. The recovery time of H_2S adsorbed on Cu-SnP_3 at 298 K, 398 K, and 498 K, respectively.
